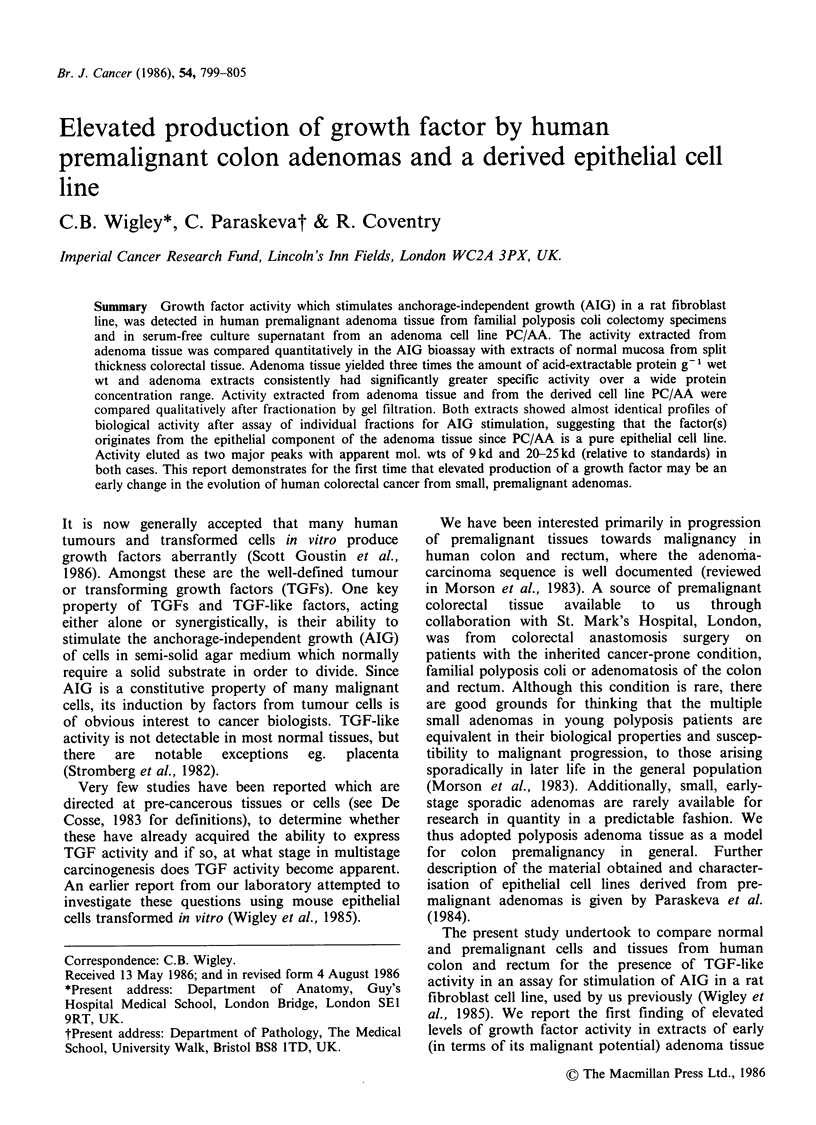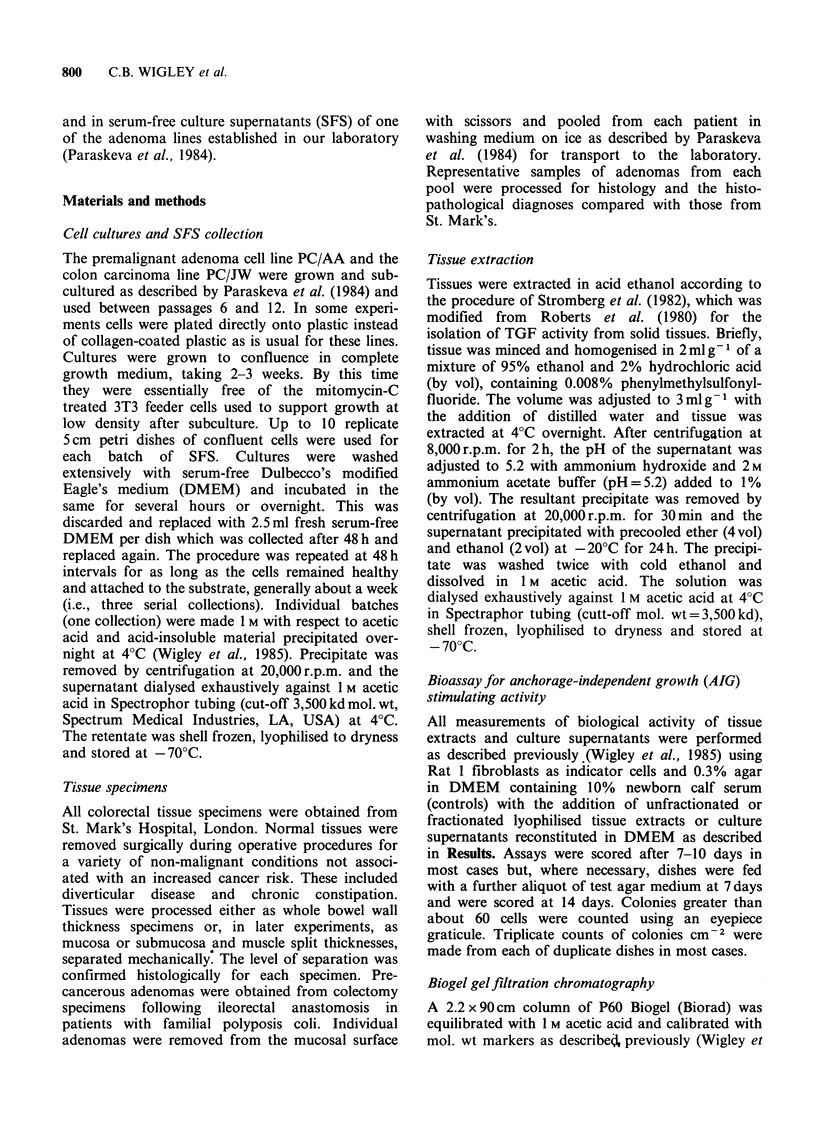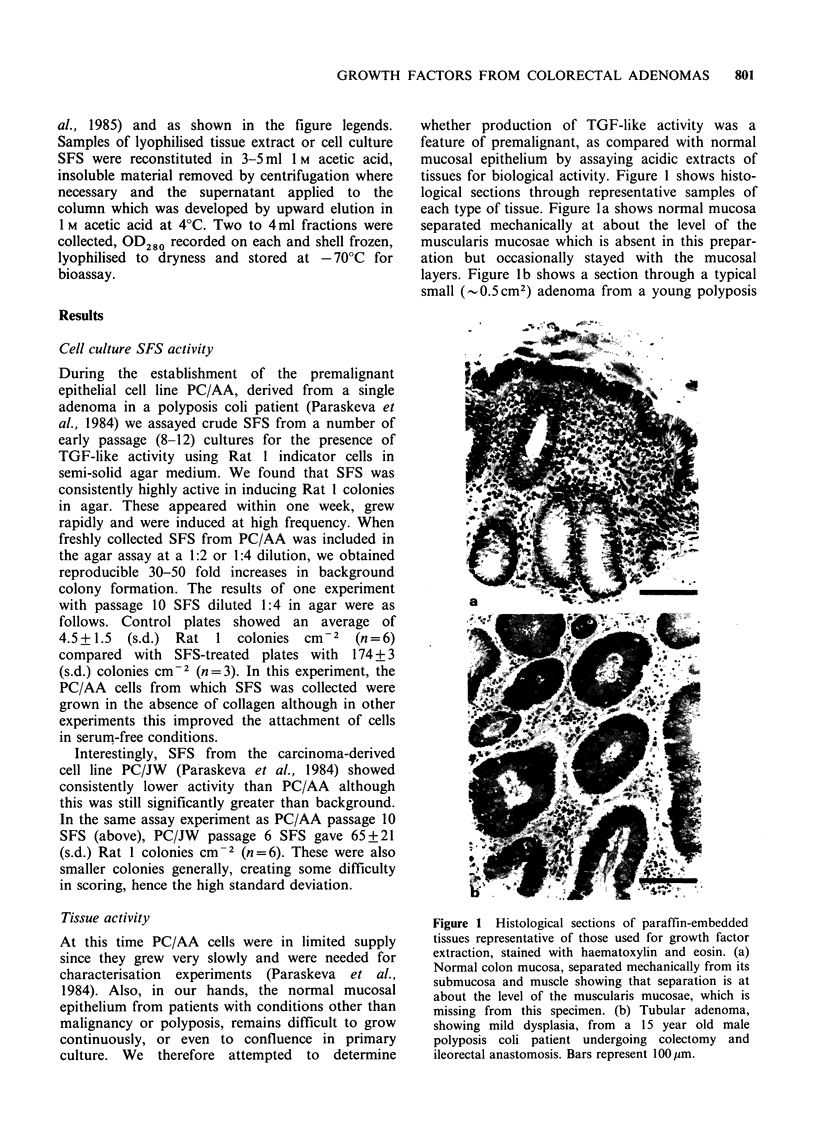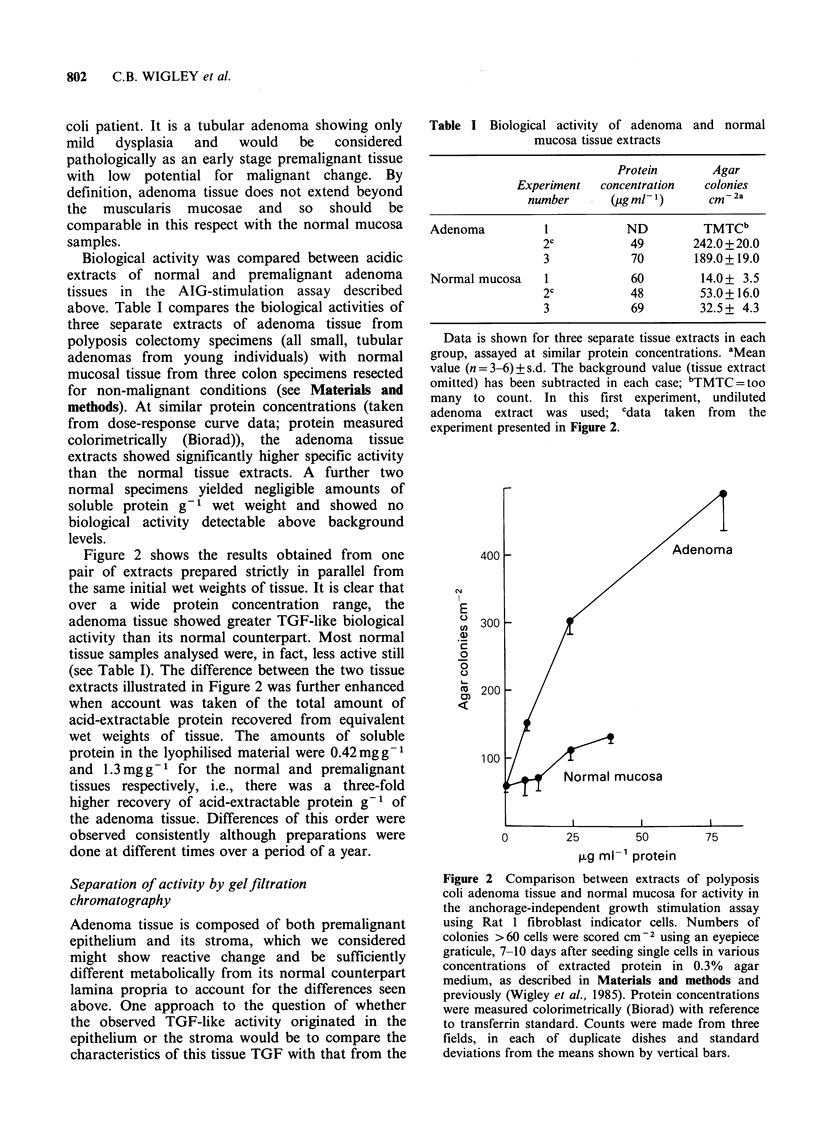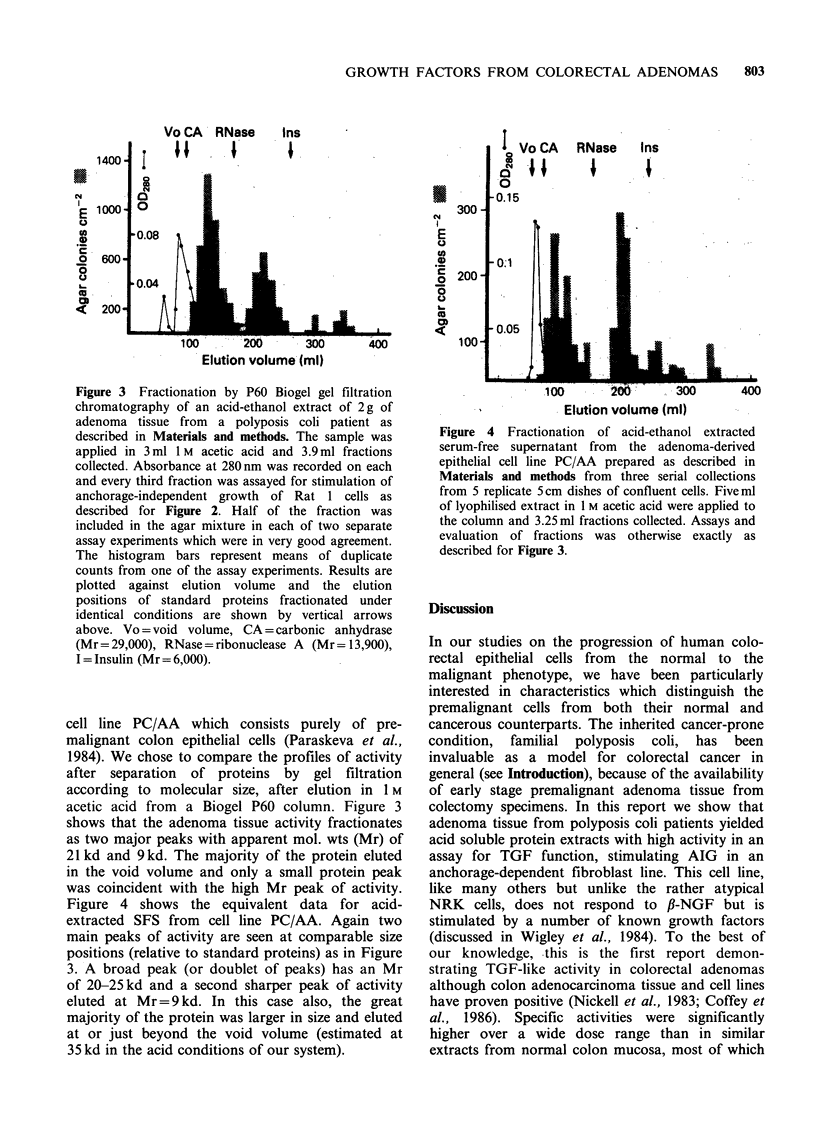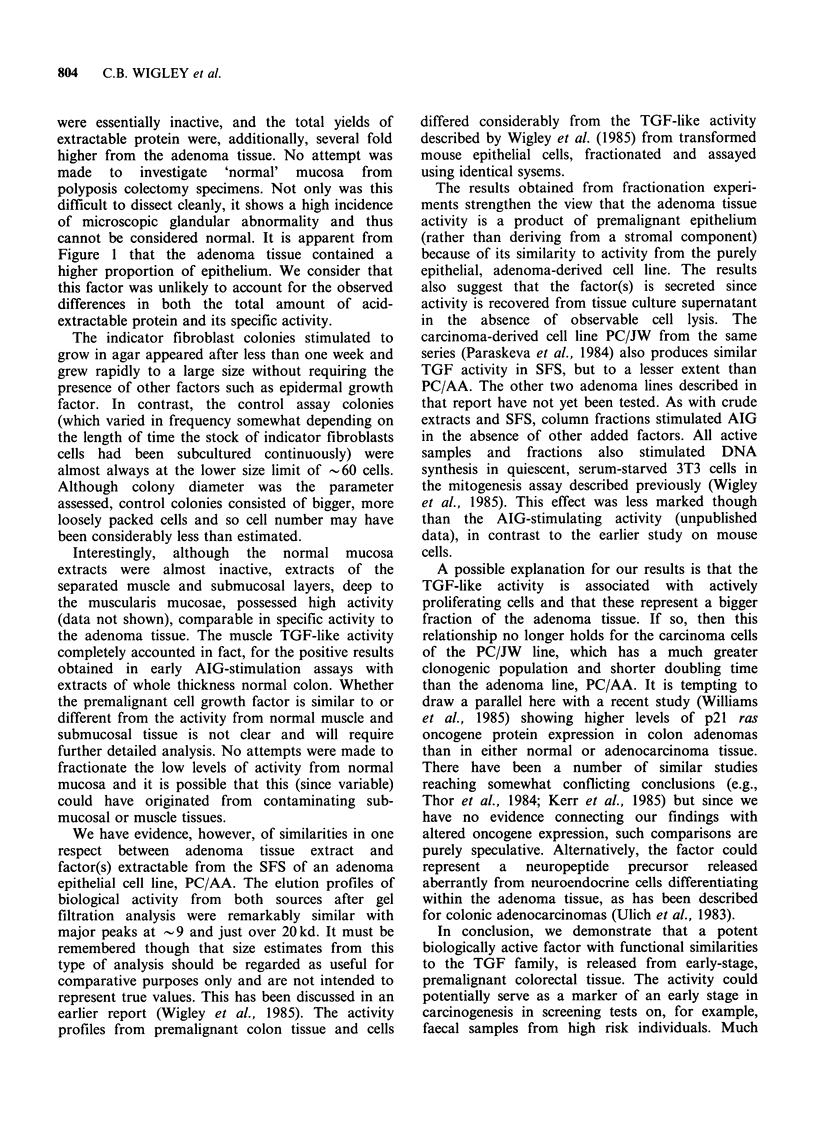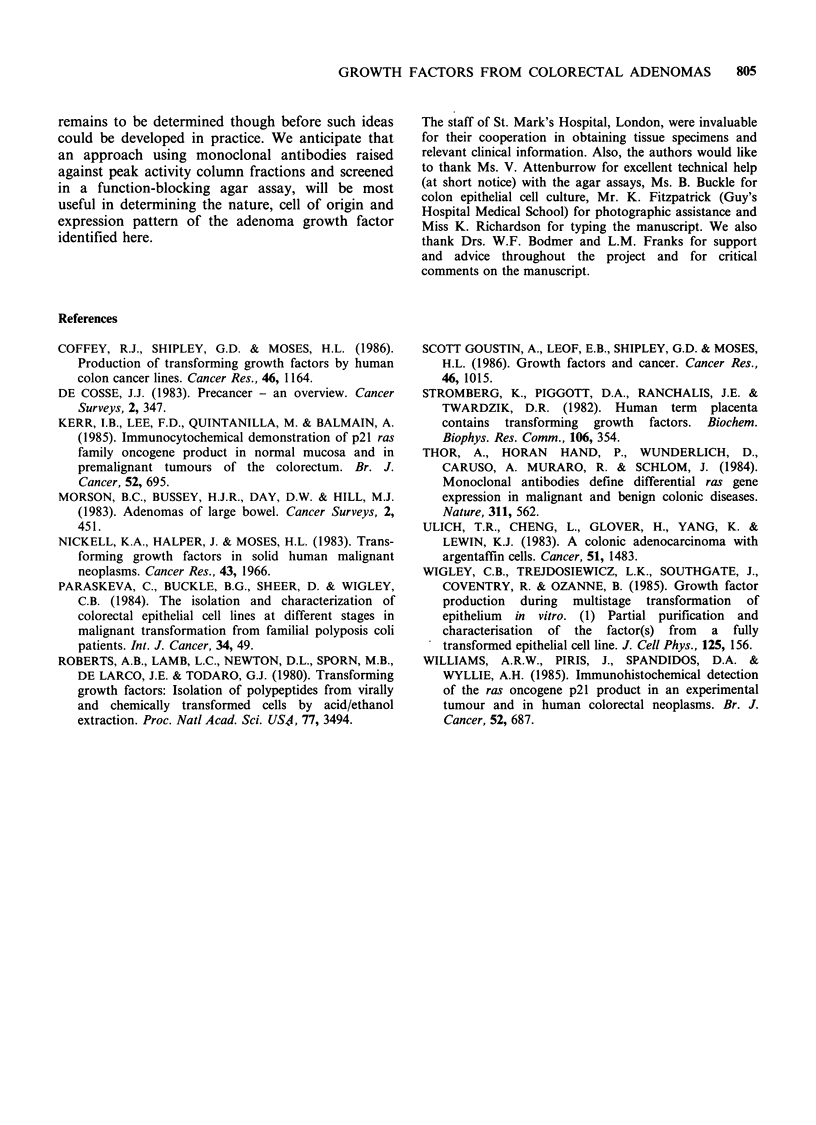# Elevated production of growth factor by human premalignant colon adenomas and a derived epithelial cell line.

**DOI:** 10.1038/bjc.1986.243

**Published:** 1986-11

**Authors:** C. B. Wigley, C. Paraskeva, R. Coventry

## Abstract

**Images:**


*Br. J. Cancer* (1986), **54**, 799–805

### Elevated production of growth factor by human premalignant colon adenomas and a derived epithelial cell line

C.B. Wigley*, C. Paraskeva† & R. Coventry

*Imperial Cancer Research Fund, Lincoln's Inn Fields, London WC2A 3PX, UK.*

**Summary** Growth factor activity which stimulates anchorage-independent growth (AIG) in a rat fibroblast line, was detected in human premalignant adenoma tissue from familial polyposis coli colectomy specimens and in serum-free culture supernatant from an adenoma cell line PC/AA. The activity extracted from adenoma tissue was compared quantitatively in the AIG bioassay with extracts of normal mucosa from split thickness colorectal tissue. Adenoma tissue yielded three times the amount of acid-extractable protein $g^{-1}$ wet wt and adenoma extracts consistently had significantly greater specific activity over a wide protein concentration range. Activity extracted from adenoma tissue and from the derived cell line PC/AA were compared qualitatively after fractionation by gel filtration. Both extracts showed almost identical profiles of biological activity after assay of individual fractions for AIG stimulation, suggesting that the factor(s) originates from the epithelial component of the adenoma tissue since PC/AA is a pure epithelial cell line. Activity eluted as two major peaks with apparent mol. wts of 9 kd and 20–25 kd (relative to standards) in both cases. This report demonstrates for the first time that elevated production of a growth factor may be an early change in the evolution of human colorectal cancer from small, premalignant adenomas.

It is now generally accepted that many human tumours and transformed cells *in vitro* produce growth factors aberrantly (Scott Goustin *et al.*, 1986). Amongst these are the well-defined tumour or transforming growth factors (TGFs). One key property of TGFs and TGF-like factors, acting either alone or synergistically, is their ability to stimulate the anchorage-independent growth (AIG) of cells in semi-solid agar medium which normally require a solid substrate in order to divide. Since AIG is a constitutive property of many malignant cells, its induction by factors from tumour cells is of obvious interest to cancer biologists. TGF-like activity is not detectable in most normal tissues, but there are notable exceptions eg. placenta (Stromberg *et al.*, 1982).

Very few studies have been reported which are directed at pre-cancerous tissues or cells (see De Cosse, 1983 for definitions), to determine whether these have already acquired the ability to express TGF activity and if so, at what stage in multistage carcinogenesis does TGF activity become apparent. An earlier report from our laboratory attempted to investigate these questions using mouse epithelial cells transformed *in vitro* (Wigley *et al.*, 1985).

Correspondence: C.B. Wigley.
Received 13 May 1986; and in revised form 4 August 1986
*Present address: Department of Anatomy, Guy's Hospital Medical School, London Bridge, London SE1 9RT, UK.
†Present address: Department of Pathology, The Medical School, University Walk, Bristol BS8 1TD, UK.

We have been interested primarily in progression of premalignant tissues towards malignancy in human colon and rectum, where the adenoma-carcinoma sequence is well documented (reviewed in Morson *et al.*, 1983). A source of premalignant colorectal tissue available to us through collaboration with St. Mark's Hospital, London, was from colorectal anastomosis surgery on patients with the inherited cancer-prone condition, familial polyposis coli or adenomatosis of the colon and rectum. Although this condition is rare, there are good grounds for thinking that the multiple small adenomas in young polyposis patients are equivalent in their biological properties and susceptibility to malignant progression, to those arising sporadically in later life in the general population (Morson *et al.*, 1983). Additionally, small, early-stage sporadic adenomas are rarely available for research in quantity in a predictable fashion. We thus adopted polyposis adenoma tissue as a model for colon premalignancy in general. Further description of the material obtained and characterisation of epithelial cell lines derived from premalignant adenomas is given by Paraskeva *et al.* (1984).

The present study undertook to compare normal and premalignant cells and tissues from human colon and rectum for the presence of TGF-like activity in an assay for stimulation of AIG in a rat fibroblast cell line, used by us previously (Wigley *et al.*, 1985). We report the first finding of elevated levels of growth factor activity in extracts of early (in terms of its malignant potential) adenoma tissue

© The Macmillan Press Ltd., 1986

and in serum-free culture supernatants (SFS) of one of the adenoma lines established in our laboratory (Paraskeva *et al.*, 1984).

### Materials and methods

### *Cell cultures and SFS collection*

The premalignant adenoma cell line PC/AA and the colon carcinoma line PC/JW were grown and subcultured as described by Paraskeva *et al.* (1984) and used between passages 6 and 12. In some experiments cells were plated directly onto plastic instead of collagen-coated plastic as is usual for these lines. Cultures were grown to confluence in complete growth medium, taking 2–3 weeks. By this time they were essentially free of the mitomycin-C treated 3T3 feeder cells used to support growth at low density after subculture. Up to 10 replicate 5 cm petri dishes of confluent cells were used for each batch of SFS. Cultures were washed extensively with serum-free Dulbecco's modified Eagle's medium (DMEM) and incubated in the same for several hours or overnight. This was discarded and replaced with 2.5 ml fresh serum-free DMEM per dish which was collected after 48 h and replaced again. The procedure was repeated at 48 h intervals for as long as the cells remained healthy and attached to the substrate, generally about a week (i.e., three serial collections). Individual batches (one collection) were made 1 M with respect to acetic acid and acid-insoluble material precipitated overnight at 4°C (Wigley *et al.*, 1985). Precipitate was removed by centrifugation at 20,000 r.p.m. and the supernatant dialysed exhaustively against 1 M acetic acid in Spectrophor tubing (cut-off 3,500 kd mol. wt, Spectrum Medical Industries, LA, USA) at 4°C. The retentate was shell frozen, lyophilised to dryness and stored at −70°C.

### *Tissue specimens*

All colorectal tissue specimens were obtained from St. Mark's Hospital, London. Normal tissues were removed surgically during operative procedures for a variety of non-malignant conditions not associated with an increased cancer risk. These included diverticular disease and chronic constipation. Tissues were processed either as whole bowel wall thickness specimens or, in later experiments, as mucosa or submucosa and muscle split thicknesses, separated mechanically. The level of separation was confirmed histologically for each specimen. Precancerous adenomas were obtained from colectomy specimens following ileorectal anastomosis in patients with familial polyposis coli. Individual adenomas were removed from the mucosal surface with scissors and pooled from each patient in washing medium on ice as described by Paraskeva *et al.* (1984) for transport to the laboratory. Representative samples of adenomas from each pool were processed for histology and the histopathological diagnoses compared with those from St. Mark's.

### *Tissue extraction*

Tissues were extracted in acid ethanol according to the procedure of Stromberg *et al.* (1982), which was modified from Roberts *et al.* (1980) for the isolation of TGF activity from solid tissues. Briefly, tissue was minced and homogenised in 2 ml g$^{-1}$ of a mixture of 95% ethanol and 2% hydrochloric acid (by vol), containing 0.008% phenylmethylsulfonylfluoride. The volume was adjusted to 3 ml g$^{-1}$ with the addition of distilled water and tissue was extracted at 4°C overnight. After centrifugation at 8,000 r.p.m. for 2 h, the pH of the supernatant was adjusted to 5.2 with ammonium hydroxide and 2 M ammonium acetate buffer (pH = 5.2) added to 1% (by vol). The resultant precipitate was removed by centrifugation at 20,000 r.p.m. for 30 min and the supernatant precipitated with precooled ether (4 vol) and ethanol (2 vol) at −20°C for 24 h. The precipitate was washed twice with cold ethanol and dissolved in 1 M acetic acid. The solution was dialysed exhaustively against 1 M acetic acid at 4°C in Spectraphor tubing (cutt-off mol. wt = 3,500 kd), shell frozen, lyophilised to dryness and stored at −70°C.

### *Bioassay for anchorage-independent growth (AIG) stimulating activity*

All measurements of biological activity of tissue extracts and culture supernatants were performed as described previously (Wigley *et al.*, 1985) using Rat 1 fibroblasts as indicator cells and 0.3% agar in DMEM containing 10% newborn calf serum (controls) with the addition of unfractionated or fractionated lyophilised tissue extracts or culture supernatants reconstituted in DMEM as described in **Results.** Assays were scored after 7–10 days in most cases but, where necessary, dishes were fed with a further aliquot of test agar medium at 7 days and were scored at 14 days. Colonies greater than about 60 cells were counted using an eyepiece graticule. Triplicate counts of colonies cm$^{-2}$ were made from each of duplicate dishes in most cases.

### *Biogel gel filtration chromatography*

A 2.2 × 90 cm column of P60 Biogel (Biorad) was equilibrated with 1 M acetic acid and calibrated with mol. wt markers as described previously (Wigley *et*

*al.*, 1985) and as shown in the figure legends. Samples of lyophilised tissue extract or cell culture SFS were reconstituted in 3–5 ml 1 M acetic acid, insoluble material removed by centrifugation where necessary and the supernatant applied to the column which was developed by upward elution in 1 M acetic acid at 4°C. Two to 4 ml fractions were collected, $OD_{280}$ recorded on each and shell frozen, lyophilised to dryness and stored at −70°C for bioassay.

### Results

### *Cell culture SFS activity*

During the establishment of the premalignant epithelial cell line PC/AA, derived from a single adenoma in a polyposis coli patient (Paraskeva *et al.*, 1984) we assayed crude SFS from a number of early passage (8–12) cultures for the presence of TGF-like activity using Rat 1 indicator cells in semi-solid agar medium. We found that SFS was consistently highly active in inducing Rat 1 colonies in agar. These appeared within one week, grew rapidly and were induced at high frequency. When freshly collected SFS from PC/AA was included in the agar assay at a 1:2 or 1:4 dilution, we obtained reproducible 30–50 fold increases in background colony formation. The results of one experiment with passage 10 SFS diluted 1:4 in agar were as follows. Control plates showed an average of $4.5 \pm 1.5$ (s.d.) Rat 1 colonies $cm^{-2}$ ($n=6$) compared with SFS-treated plates with $174 \pm 3$ (s.d.) colonies $cm^{-2}$ ($n=3$). In this experiment, the PC/AA cells from which SFS was collected were grown in the absence of collagen although in other experiments this improved the attachment of cells in serum-free conditions.

Interestingly, SFS from the carcinoma-derived cell line PC/JW (Paraskeva *et al.*, 1984) showed consistently lower activity than PC/AA although this was still significantly greater than background. In the same assay experiment as PC/AA passage 10 SFS (above), PC/JW passage 6 SFS gave $65 \pm 21$ (s.d.) Rat 1 colonies $cm^{-2}$ ($n=6$). These were also smaller colonies generally, creating some difficulty in scoring, hence the high standard deviation.

### *Tissue activity*

At this time PC/AA cells were in limited supply since they grew very slowly and were needed for characterisation experiments (Paraskeva *et al.*, 1984). Also, in our hands, the normal mucosal epithelium from patients with conditions other than malignancy or polyposis, remains difficult to grow continuously, or even to confluence in primary culture. We therefore attempted to determine whether production of TGF-like activity was a feature of premalignant, as compared with normal mucosal epithelium by assaying acidic extracts of tissues for biological activity. Figure 1 shows histological sections through representative samples of each type of tissue. Figure 1a shows normal mucosa separated mechanically at about the level of the muscularis mucosae which is absent in this preparation but occasionally stayed with the mucosal layers. Figure 1b shows a section through a typical small (~0.5 $cm^2$) adenoma from a young polyposis

**Figure 1** Histological sections of paraffin-embedded tissues representative of those used for growth factor extraction, stained with haematoxylin and eosin. (a) Normal colon mucosa, separated mechanically from its submucosa and muscle showing that separation is at about the level of the muscularis mucosae, which is missing from this specimen. (b) Tubular adenoma, showing mild dysplasia, from a 15 year old male polyposis coli patient undergoing colectomy and ileorectal anastomosis. Bars represent 100 μm.

coli patient. It is a tubular adenoma showing only mild dysplasia and would be considered pathologically as an early stage premalignant tissue with low potential for malignant change. By definition, adenoma tissue does not extend beyond the muscularis mucosae and so should be comparable in this respect with the normal mucosa samples.

Biological activity was compared between acidic extracts of normal and premalignant adenoma tissues in the AIG-stimulation assay described above. Table I compares the biological activities of three separate extracts of adenoma tissue from polyposis colectomy specimens (all small, tubular adenomas from young individuals) with normal mucosal tissue from three colon specimens resected for non-malignant conditions (see **Materials and methods**). At similar protein concentrations (taken from dose-response curve data; protein measured colorimetrically (Biorad)), the adenoma tissue extracts showed significantly higher specific activity than the normal tissue extracts. A further two normal specimens yielded negligible amounts of soluble protein $g^{-1}$ wet weight and showed no biological activity detectable above background levels.

Figure 2 shows the results obtained from one pair of extracts prepared strictly in parallel from the same initial wet weights of tissue. It is clear that over a wide protein concentration range, the adenoma tissue showed greater TGF-like biological activity than its normal counterpart. Most normal tissue samples analysed were, in fact, less active still (see Table I). The difference between the two tissue extracts illustrated in Figure 2 was further enhanced when account was taken of the total amount of acid-extractable protein recovered from equivalent wet weights of tissue. The amounts of soluble protein in the lyophilised material were $0.42\,mg\,g^{-1}$ and $1.3\,mg\,g^{-1}$ for the normal and premalignant tissues respectively, i.e., there was a three-fold higher recovery of acid-extractable protein $g^{-1}$ of the adenoma tissue. Differences of this order were observed consistently although preparations were done at different times over a period of a year.

*Separation of activity by gel filtration chromatography*

Adenoma tissue is composed of both premalignant epithelium and its stroma, which we considered might show reactive change and be sufficiently different metabolically from its normal counterpart lamina propria to account for the differences seen above. One approach to the question of whether the observed TGF-like activity originated in the epithelium or the stroma would be to compare the characteristics of this tissue TGF with that from the

**Table I** Biological activity of adenoma and normal mucosa tissue extracts

| | *Experiment number* | *Protein concentration* ($\mu g\,ml^{-1}$) | *Agar colonies* $cm^{-2a}$ |
|---|---|---|---|
| Adenoma | 1 | ND | TMTC[b] |
| | 2[c] | 49 | $242.0 \pm 20.0$ |
| | 3 | 70 | $189.0 \pm 19.0$ |
| Normal mucosa | 1 | 60 | $14.0 \pm 3.5$ |
| | 2[c] | 48 | $53.0 \pm 16.0$ |
| | 3 | 69 | $32.5 \pm 4.3$ |

Data is shown for three separate tissue extracts in each group, assayed at similar protein concentrations. [a]Mean value ($n = 3$–$6$) $\pm$ s.d. The background value (tissue extract omitted) has been subtracted in each case; [b]TMTC = too many to count. In this first experiment, undiluted adenoma extract was used; [c]data taken from the experiment presented in **Figure 2**.

**Figure 2** Comparison between extracts of polyposis coli adenoma tissue and normal mucosa for activity in the anchorage-independent growth stimulation assay using Rat 1 fibroblast indicator cells. Numbers of colonies >60 cells were scored $cm^{-2}$ using an eyepiece graticule, 7–10 days after seeding single cells in various concentrations of extracted protein in 0.3% agar medium, as described in **Materials and methods** and previously (Wigley *et al.*, 1985). Protein concentrations were measured colorimetrically (Biorad) with reference to transferrin standard. Counts were made from three fields, in each of duplicate dishes and standard deviations from the means shown by vertical bars.

**Figure 3** Fractionation by P60 Biogel gel filtration chromatography of an acid-ethanol extract of 2 g of adenoma tissue from a polyposis coli patient as described in **Materials and methods.** The sample was applied in 3 ml 1 M acetic acid and 3.9 ml fractions collected. Absorbance at 280 nm was recorded on each and every third fraction was assayed for stimulation of anchorage-independent growth of Rat 1 cells as described for **Figure 2**. Half of the fraction was included in the agar mixture in each of two separate assay experiments which were in very good agreement. The histogram bars represent means of duplicate counts from one of the assay experiments. Results are plotted against elution volume and the elution positions of standard proteins fractionated under identical conditions are shown by vertical arrows above. Vo = void volume, CA = carbonic anhydrase (Mr = 29,000), RNase = ribonuclease A (Mr = 13,900), I = Insulin (Mr = 6,000).

**Figure 4** Fractionation of acid-ethanol extracted serum-free supernatant from the adenoma-derived epithelial cell line PC/AA prepared as described in **Materials and methods** from three serial collections from 5 replicate 5 cm dishes of confluent cells. Five ml of lyophilised extract in 1 M acetic acid were applied to the column and 3.25 ml fractions collected. Assays and evaluation of fractions was otherwise exactly as described for **Figure 3**.

cell line PC/AA which consists purely of premalignant colon epithelial cells (Paraskeva *et al.*, 1984). We chose to compare the profiles of activity after separation of proteins by gel filtration according to molecular size, after elution in 1 M acetic acid from a Biogel P60 column. Figure 3 shows that the adenoma tissue activity fractionates as two major peaks with apparent mol. wts (Mr) of 21 kd and 9 kd. The majority of the protein eluted in the void volume and only a small protein peak was coincident with the high Mr peak of activity. Figure 4 shows the equivalent data for acid-extracted SFS from cell line PC/AA. Again two main peaks of activity are seen at comparable size positions (relative to standard proteins) as in Figure 3. A broad peak (or doublet of peaks) has an Mr of 20–25 kd and a second sharper peak of activity eluted at Mr = 9 kd. In this case also, the great majority of the protein was larger in size and eluted at or just beyond the void volume (estimated at 35 kd in the acid conditions of our system).

### Discussion

In our studies on the progression of human colorectal epithelial cells from the normal to the malignant phenotype, we have been particularly interested in characteristics which distinguish the premalignant cells from both their normal and cancerous counterparts. The inherited cancer-prone condition, familial polyposis coli, has been invaluable as a model for colorectal cancer in general (see **Introduction**), because of the availability of early stage premalignant adenoma tissue from colectomy specimens. In this report we show that adenoma tissue from polyposis coli patients yielded acid soluble protein extracts with high activity in an assay for TGF function, stimulating AIG in an anchorage-dependent fibroblast line. This cell line, like many others but unlike the rather atypical NRK cells, does not respond to $\beta$-NGF but is stimulated by a number of known growth factors (discussed in Wigley *et al.*, 1984). To the best of our knowledge, this is the first report demonstrating TGF-like activity in colorectal adenomas although colon adenocarcinoma tissue and cell lines have proven positive (Nickell *et al.*, 1983; Coffey *et al.*, 1986). Specific activities were significantly higher over a wide dose range than in similar extracts from normal colon mucosa, most of which were essentially inactive, and the total yields of extractable protein were, additionally, several fold higher from the adenoma tissue. No attempt was made to investigate 'normal' mucosa from polyposis colectomy specimens. Not only was this difficult to dissect cleanly, it shows a high incidence of microscopic glandular abnormality and thus cannot be considered normal. It is apparent from Figure 1 that the adenoma tissue contained a higher proportion of epithelium. We consider that this factor was unlikely to account for the observed differences in both the total amount of acid-extractable protein and its specific activity.

The indicator fibroblast colonies stimulated to grow in agar appeared after less than one week and grew rapidly to a large size without requiring the presence of other factors such as epidermal growth factor. In contrast, the control assay colonies (which varied in frequency somewhat depending on the length of time the stock of indicator fibroblasts cells had been subcultured continuously) were almost always at the lower size limit of $\sim$60 cells. Although colony diameter was the parameter assessed, control colonies consisted of bigger, more loosely packed cells and so cell number may have been considerably less than estimated.

Interestingly, although the normal mucosa extracts were almost inactive, extracts of the separated muscle and submucosal layers, deep to the muscularis mucosae, possessed high activity (data not shown), comparable in specific activity to the adenoma tissue. The muscle TGF-like activity completely accounted in fact, for the positive results obtained in early AIG-stimulation assays with extracts of whole thickness normal colon. Whether the premalignant cell growth factor is similar to or different from the activity from normal muscle and submucosal tissue is not clear and will require further detailed analysis. No attempts were made to fractionate the low levels of activity from normal mucosa and it is possible that this (since variable) could have originated from contaminating submucosal or muscle tissues.

We have evidence, however, of similarities in one respect between adenoma tissue extract and factor(s) extractable from the SFS of an adenoma epithelial cell line, PC/AA. The elution profiles of biological activity from both sources after gel filtration analysis were remarkably similar with major peaks at $\sim$9 and just over 20 kd. It must be remembered though that size estimates from this type of analysis should be regarded as useful for comparative purposes only and are not intended to represent true values. This has been discussed in an earlier report (Wigley *et al.*, 1985). The activity profiles from premalignant colon tissue and cells differed considerably from the TGF-like activity described by Wigley *et al.* (1985) from transformed mouse epithelial cells, fractionated and assayed using identical sysems.

The results obtained from fractionation experiments strengthen the view that the adenoma tissue activity is a product of premalignant epithelium (rather than deriving from a stromal component) because of its similarity to activity from the purely epithelial, adenoma-derived cell line. The results also suggest that the factor(s) is secreted since activity is recovered from tissue culture supernatant in the absence of observable cell lysis. The carcinoma-derived cell line PC/JW from the same series (Paraskeva *et al.*, 1984) also produces similar TGF activity in SFS, but to a lesser extent than PC/AA. The other two adenoma lines described in that report have not yet been tested. As with crude extracts and SFS, column fractions stimulated AIG in the absence of other added factors. All active samples and fractions also stimulated DNA synthesis in quiescent, serum-starved 3T3 cells in the mitogenesis assay described previously (Wigley *et al.*, 1985). This effect was less marked though than the AIG-stimulating activity (unpublished data), in contrast to the earlier study on mouse cells.

A possible explanation for our results is that the TGF-like activity is associated with actively proliferating cells and that these represent a bigger fraction of the adenoma tissue. If so, then this relationship no longer holds for the carcinoma cells of the PC/JW line, which has a much greater clonogenic population and shorter doubling time than the adenoma line, PC/AA. It is tempting to draw a parallel here with a recent study (Williams *et al.*, 1985) showing higher levels of p21 *ras* oncogene protein expression in colon adenomas than in either normal or adenocarcinoma tissue. There have been a number of similar studies reaching somewhat conflicting conclusions (e.g., Thor *et al.*, 1984; Kerr *et al.*, 1985) but since we have no evidence connecting our findings with altered oncogene expression, such comparisons are purely speculative. Alternatively, the factor could represent a neuropeptide precursor released aberrantly from neuroendocrine cells differentiating within the adenoma tissue, as has been described for colonic adenocarcinomas (Ulich *et al.*, 1983).

In conclusion, we demonstrate that a potent biologically active factor with functional similarities to the TGF family, is released from early-stage, premalignant colorectal tissue. The activity could potentially serve as a marker of an early stage in carcinogenesis in screening tests on, for example, faecal samples from high risk individuals. Much remains to be determined though before such ideas could be developed in practice. We anticipate that an approach using monoclonal antibodies raised against peak activity column fractions and screened in a function-blocking agar assay, will be most useful in determining the nature, cell of origin and expression pattern of the adenoma growth factor identified here.

The staff of St. Mark's Hospital, London, were invaluable for their cooperation in obtaining tissue specimens and relevant clinical information. Also, the authors would like to thank Ms. V. Attenburrow for excellent technical help (at short notice) with the agar assays, Ms. B. Buckle for colon epithelial cell culture, Mr. K. Fitzpatrick (Guy's Hospital Medical School) for photographic assistance and Miss K. Richardson for typing the manuscript. We also thank Drs. W.F. Bodmer and L.M. Franks for support and advice throughout the project and for critical comments on the manuscript.

### References

COFFEY, R.J., SHIPLEY, G.D. & MOSES, H.L. (1986). Production of transforming growth factors by human colon cancer lines. *Cancer Res.*, **46**, 1164.

DE COSSE, J.J. (1983). Precancer – an overview. *Cancer Surveys*, **2**, 347.

KERR, I.B., LEE, F.D., QUINTANILLA, M. & BALMAIN, A. (1985). Immunocytochemical demonstration of p21 *ras* family oncogene product in normal mucosa and in premalignant tumours of the colorectum. *Br. J. Cancer*, **52**, 695.

MORSON, B.C., BUSSEY, H.J.R., DAY, D.W. & HILL, M.J. (1983). Adenomas of large bowel. *Cancer Surveys*, **2**, 451.

NICKELL, K.A., HALPER, J. & MOSES, H.L. (1983). Transforming growth factors in solid human malignant neoplasms. *Cancer Res.*, **43**, 1966.

PARASKEVA, C., BUCKLE, B.G., SHEER, D. & WIGLEY, C.B. (1984). The isolation and characterization of colorectal epithelial cell lines at different stages in malignant transformation from familial polyposis coli patients. *Int. J. Cancer*, **34**, 49.

ROBERTS, A.B., LAMB, L.C., NEWTON, D.L., SPORN, M.B., DE LARCO, J.E. & TODARO, G.J. (1980). Transforming growth factors: Isolation of polypeptides from virally and chemically transformed cells by acid/ethanol extraction. *Proc. Natl Acad. Sci. USA*, **77**, 3494.

SCOTT GOUSTIN, A., LEOF, E.B., SHIPLEY, G.D. & MOSES, H.L. (1986). Growth factors and cancer. *Cancer Res.*, **46**, 1015.

STROMBERG, K., PIGGOTT, D.A., RANCHALIS, J.E. & TWARDZIK, D.R. (1982). Human term placenta contains transforming growth factors. *Biochem. Biophys. Res. Comm.*, **106**, 354.

THOR, A., HORAN HAND, P., WUNDERLICH, D., CARUSO, A. MURARO, R. & SCHLOM, J. (1984). Monoclonal antibodies define differential *ras* gene expression in malignant and benign colonic diseases. *Nature*, **311**, 562.

ULICH, T.R., CHENG, L., GLOVER, H., YANG, K. & LEWIN, K.J. (1983). A colonic adenocarcinoma with argentaffin cells. *Cancer*, **51**, 1483.

WIGLEY, C.B., TREJDOSIEWICZ, L.K., SOUTHGATE, J., COVENTRY, R. & OZANNE, B. (1985). Growth factor production during multistage transformation of epithelium *in vitro*. (1) Partial purification and characterisation of the factor(s) from a fully transformed epithelial cell line. *J. Cell Phys.*, **125**, 156.

WILLIAMS, A.R.W., PIRIS, J., SPANDIDOS, D.A. & WYLLIE, A.H. (1985). Immunohistochemical detection of the *ras* oncogene p21 product in an experimental tumour and in human colorectal neoplasms. *Br. J. Cancer*, **52**, 687.